# Metabolic reprogramming in cardiac fibrosis: mechanisms, crosstalk, and therapeutic interventions

**DOI:** 10.3389/fphys.2026.1811882

**Published:** 2026-07-27

**Authors:** Xiaoqian Wang, Xiaomei Wang, Fei Guo, Qian Ding, Honghua Jin

**Affiliations:** 1 Department of Pharmacy, The Affiliated Hospital of Yanbian University (Yanbian Hospital), Yanbian University, Yanji, China; 2 Shanghai Fourth People's Hospital, School of Medicine, Tongji University, Shanghai, China; 3 College of Pharmacy, Yanbian University, Yanji, China

**Keywords:** amino acid metabolism, cardiac fibrosis, glycolysis, lipid metabolism, metabolic reprogramming

## Abstract

Cardiac fibrosis is a pathological process accompanied by the development of cardiovascular diseases, which is mainly manifested as excessive deposition of extracellular matrix (ECM) of the heart muscle. Recent studies indicate that metabolic reprogramming significantly influences fibroblast activation, collagen deposition, and the fibrotic process by reshaping glycolytic, lipid, and amino acid metabolic pathways. This paper systematically reviews the mechanism of metabolic reprogramming in cardiac fibrosis and puts forward potential intervention strategies from a critical perspective. Although metabolic reprogramming shows potential in regulating cardiac fibrosis, its practical application still faces major challenges. Future research should strive to build a comprehensive research framework and promote metabolic reprogramming research to open a new way of precise intervention for the prevention and treatment of cardiac fibrosis through cross−technological innovation and precise regulation.

## 1 Introduction

Cardiovascular disease is an important problem affecting the health of millions of people around the world. Cardiac fibrosis is caused by myocardial fiber dilation caused by myocardial extracellular matrix (ECM) protein deposition, which is a variety of characteristic changes in the end-stage heart disease, such as myocardial infarction (MI), heart failure and myocardial hypertrophy (Travers et al., 2016). MI is usually caused by the rupture of the inner wall of the blood vessel to form a thrombus blocking the coronary artery, and the regeneration ability of adult myocardial cells is weak. Myocardial fibroblasts are responsible for the production of collagen and are also the main source of matrix protein (Tallquist, 2020). After MI, cardiac fibroblasts proliferate and differentiate into myofibroblasts, synthesizing ECM components, thus promoting the formation of scar tissue. The damaged myocardial tissue is replaced by these scar tissues to maintain the integrity of the chamber structure. Currently, heart fibrosis plays a restorative role. However, excessive fibrosis is harmful, leading to gradual impairment of heart function, aggravation of diastolic dysfunction, and ultimately leading to heart failure (Ye et al., 2023).

Metabolic reprogramming is the regulation of cells to adapt to the environment to meet their own energy needs. When cells are pathologically stimulated, their original metabolic balance will be broken, involving the reshaping of glucose, lipids, amino acids and other metabolic pathways to meet the new needs of cell proliferation, survival and functional change (Tani et al., 2023). The normal heart mainly relies on the oxidative phosphorylation (OXPHOS) of fatty acids to generate energy, and uses other metabolites, such as glucose and amino acids. When oxygen or nutrition is insufficient in the environment, myocardial fibroblasts must reprogram their metabolic pathways to meet their own energy, biosynthesis and redox needs (Wu H. et al., 2023).

At present, there are several ways to treat or reverse cardiac fibrosis. For example, after stem cell therapy is applied to cardiac fibrosis, it shows a low implantation rate and survival rate; new mediators to relieve cardiac fibrosis such as miRNA and cytokines have been used in clinical experiments, and the results are disappointing. Even though drugs like renin-angiotensin-aldosterone system have been proven to have clinical efficacy, the extent of cardiac fibrosis has only shown a certain degree of slowing (Fang et al., 2017). Metabolic reprogramming of myocardial fibroblasts may be able to effectively inhibit the excessive deposition of ECM, to achieve effective treatment (Liu M. et al., 2021).

This article focuses on the importance of metabolic reprogramming in the development of cardiac fibrosis, and explores treatment strategies from the perspective of glucose, lipid, and amino acid metabolism reprogramming.

## 2 Myocardial metabolic reprogramming

Of the ATP produced by the heart, approximately 95% comes from mitochondrial OXPHOS, and only a small fraction from glycolysis under physiological conditions (Da Dalt et al., 2023). Normal myocardial cells mainly rely on aerobic oxidation-based energy metabolism patterns to produce a large amount of adenosine triphosphate (ATP) needed to maintain cardiac contraction and basal metabolism through mitochondria. The ATP produced by mitochondria mainly comes from the oxidation of free fatty acids and glucose, followed by lactic acid, ketoacids, and pyruvate (Lopaschuk et al., 2021). The contraction and diastole of the heart muscle is an energy-consuming process, and ATP is the only available form of energy for the heart muscle. In normal physiological conditions, cardiac metabolism is completed by glucose and free fatty acids. There are two main metabolic pathways of glucose in the heart: anaerobic fermentation and aerobic oxidation, which provide energy for the synthesis of biological macromolecules through the pentose phosphate route for cell growth (Chen et al., 2023). After free fatty acids are ingested in myocardial cells, they enter the mitochondria through carnitine palmityl transferase 1 (CPT1) and II and generate ATP through the β-oxidation process to maintain the function of myocardial cells (Da Dalt et al., 2023) (Li AL. et al., 2023). Leucine, isoleucine, and other amino acids can be directly oxidized within cardiomyocytes, entering the tricarboxylic acid (TCA) cycle to generate ATP. Amino acid metabolism plays an important role in the heart muscle. For example, arginine is crucial to the proliferation of fibroblasts and the synthesis of ECM components, and glutamine (Gln) is an essential substrate for cell metabolism and participates in the growth and metabolic activities of fibroblasts (Yang et al., 2024). In the pathological process of cardiac fibrosis, the metabolic mode of myocardial cells and fibroblasts has undergone significant changes, such as changing from dependent fatty acid oxidation (FAO) to glycolysis enhancement, abnormal metabolism of lipids and amino acids. These changes not only affect energy supply, but also directly regulate the fibrosis process through signaling pathways and metabolites (Sun et al., 2024). Reprogramming the energy metabolism of myocardial cells may promote myocardial reconstruction, thus playing a positive role in treatments targeting cardiac fibrosis. The interplay between amino acid, glucose and lipid metabolism, which complement each other, plays a synergistic role in myocardial function.

## 3 Specific manifestations of metabolic reprogramming in cardiac fibrosis

### 3.1 Amino acid metabolism reprogramming

Amino acids can be divided into two categories according to their metabolic characteristics: non-essential amino acids (such as glutamic acid) and essential amino acids (such as leucine). Recent studies have shown that these amino acids not only participate in the biosynthesis of proteins but also play an important regulatory role in the occurrence and development of fibrosis. Specifically, certain amino acids have been proven to affect the pathological process of fibrosis through multiple pathways, and this discovery provides a new research direction for the treatment of related diseases (Kwan et al., 2024) (Satyanarayana et al., 2021).

#### 3.1.1 Branch chain amino acid metabolism

In the process of cardiac fibrosis, the metabolic flow of Branched-chain amino acids (BCAAs) changes significantly. Its metabolites play an important role in cell signaling, protein synthesis and degradation, and have attracted much attention in recent years (Nie et al., 2018). BCAAs, including leucine, valine and isoleucine, are not only essential substrates for protein synthesis, but also play a key role in energy metabolism and signal transduction (Choi et al., 2024). The catabolism of BCAAs begins with a reversible transamination reaction. In the process, BCAAs are converted from leucine, isoleucine and valine to its branched alpha-keto acid, 2-ketoisocaproate (KIC), and 2-ketone-3-methylpentanate, 2-ketoisovalerate, respectively (Dimou et al., 2022). As a metabolite of leucine, KIC can be further converted into acetyl coenzyme A (Acetyl-CoA). Acetyl-CoA enters the nucleus through the nuclear pores, providing a substrate for the acetylation of histone and enhancing the activity of histone acetyltransferase. Histone acetylation regulates the expression of genes, which can be transcribed and activated to promote fibrosis genes, such as transforming growth factor-beta (TGF-β), Collagen type I alpha 1 (COL1A1) (He et al., 2023).

Niacinamide adenine dinucleotide (NAD^+^) is a key cofactor related to hundreds of energy metabolism processes. As NAD^+^-dependent deacetylase, Sirtuins is mainly located in the nucleus and participates in histone modification, DNA repair, cell cycle regulation and cell apoptosis process (Smirnova et al., 2024). As a member of the sirtuins family, increased evidence shows that sirtuin3 (SIRT3) plays a key role in cardiac fibrosis and even heart failure through its deacetyl modification. BCAAs metabolism can affect the activity of SIRT3 by regulating the NAD^+^ level, thus regulating histone deacetylation (such as histone H3 lysine 9) and inhibiting anti-fibrosis factors (such as nuclear factor of activated T cells 2 signaling pathway activation factor, peroxide enzyme proliferator activation receptor γ) (Peng et al., 2024) (Ning et al., 2024).

Under physiological conditions, BCAAs are first catalyzed by branched amino acid transaminase and converted into the corresponding branched-chain keto acids, and then further decomposed by the branched α-keto acid dehydrogenase complex and finally enters the TCA cycle or participates in lipid synthesis (Dimou et al., 2022). However, in the process of cardiac fibrosis, the expression and activity of BCAAs metabolic enzymes are abnormal, resulting in the accumulation of metabolic intermediates (such as α-ketoisocaproic acid and α-ketoisovaleric acid). These substances are not only intermediate products of energy metabolism but also can directly activate the signal pathway to promote fibrosis (McGarrah and White, 2023). Take KIC as an example, it can enhance the activity of the mechanistic target of rapamycin complex 1 (mTORC1), indirectly upregulate histone methyltransferase, such as enhancer of zeste homolog 2, and then catalyze the trimethylation of lysine in the 27th position of histone H3 (H3K27me3), and promote the silence of anti-fibrosis-related genes (Harachi et al., 2020). Studies show that enhancer of zeste homolog 2 and H3K27me3 play a key role in the fibrosis process. They mainly promote fibrosis through epigenetic silence of anti-fibrosis genes or activate fibrosis-promoting signaling pathways (Wu H. et al., 2023), stimulate the proliferation of myocardial fibroblasts and increase collagen synthesis, and further aggravate cardiac fibrosis (Basta et al., 2023). At the same time, the accumulation of branched-chain keto acids can increase the production of mitochondrial reactive oxygen, activate nuclear factor kappa-B (NF-κB) and TGF-β signaling pathways, and promote the development of inflammatory reactions and fibrosis (Ananieva et al., 2016).

#### 3.1.2 Glutamine metabolism and other amino acid metabolism

Gln is the most abundant amino acid in human plasma, and it is also a key substrate for cells to supplement the reactions of the TCA cycle to support mitochondrial metabolism (Liu S. et al., 2021). Gln plays a key role in metabolic reprogramming after myocardial cells are damaged. It enters the cytoplasm through transport proteins (such as solute carrier transporter 1A5 or alanine-serine-cysteine transporter 2) and supports energy metabolism through a series of reactions (Cruzat et al., 2018). Glutaminase (GLS) is a key enzyme for glutamine metabolism. GLS1 and 2 convert Gln into glutamic acid, which is then converted into α-ketoglutaric acid (α-KG). As the carbon skeleton of Gln, α-KG participates in energy metabolism through TCA cycle and can replace glucose as a key carbon source in case of ischemia or mitochondrial dysfunction (Paulusma et al., 2022). In the process of fibrosis, kinases (such as extracellular signal-regulated kinase (ERK) and mTORC1 can phosphorylate GLS, enhance its activity, promote Gln hydrolysis, and produce α-KG (Ge et al., 2018). As a cofactor for Jumonji C-family histone demethylases, α-KG directly promotes the demethylation of H3K27me3. There is evidence that demethylation of H3K27me3 can promote myofibroblast differentiation and fibrosis (Basta et al., 2023) (Han et al., 2025) (Moon et al., 2022). At the same time, the amide nitrogen resulting from GLS hydrolysis and the amino nitrogen from transamination provide nitrogen atoms necessary for the synthesis of nucleotides, amino acids (such as alanine and aspartic acid), and aminoglycoside (Thaler et al., 2022). In TGF-β1 induced fibroblasts, the expression of GLS is upregulated, resulting in enhanced degradation of Gln. This process promotes the conversion of Gln to glutamic acid, thereby improving the stability of collagen through the mechanistic target of rapamycin (mTOR) pathway, and promoting collagen deposition and fibrosis (Wu T. et al., 2023).

Myofibroblasts are the key source of cells for pathological matrix generation, which can lead to abnormal tissue structure and functional damage. Although glycolysis metabolism has multiple roles in these cells, such as providing ATP, an important function of glucose is to synthesize glycine from the beginning. By using the amino group of glutamate donors, TGF-β can induce the expression of all enzymes needed to convert glycolysis intermediate 3-phosphoglyceric acid into glycine (Darby et al., 2016). TGF-β driven collagen synthesis does not rely on exogenous glycine. Even if there is glycine in the environment, knocking out these enzymes can still significantly inhibit collagen production. This shows that myofibroblasts are more inclined to support collagen production by synthesizing glycine from the beginning on their own (Hamanaka et al., 2019). It is worth noting that amino acid metabolic reprogramming exhibits significant differences across various cardiac cell types. Cardiomyocytes, cardiac fibroblasts, and infiltrating macrophages utilize amino acids in distinct ways, which determines their different functional roles in the fibrotic process. **Table 1** (Cai et al., 2019; Liu et al., 2023; Li X. et al., 2023; Wu T. et al., 2023; Wu Y. et al., 2023; Yu et al., 2023; Bai et al., 2025) summarizes the key features of amino acid metabolism in these three major cell types and their mechanisms of fibrosis promotion.

**Table 1:** Amino acid metabolism and fibrosis in different cell types.

Cell type	Key metabolic pathways	Pro-fibrotic mechanisms	Target of intervention	References
myocardial fibroblasts	Glutamine metabolism: supporting cell proliferation and collagen formation Proline: major component of collagen, promotes ECM stability and fibrosis progression	ECM deposition and FMT	TGF-β antagonists, GLS inhibitors (such as CB-839)	(Wu T. et al., 2023) (Bai et al., 2025)
cardiomyocyte	BCAAs metabolism, aspartate metabolism, glutamate metabolism	Release of ROS pro-inflammatory factors, apoptosis/necrosis	Antioxidants (such as Nrf2), anti-apoptotic pathways (such as AKT), BCAA metabolic regulation (such as PPARα agonists)	(Yu et al., 2023) (Li X. et al., 2023)
macrophage	Arginine metabolism, tryptophan metabolism	M1/M2 polarisation, cytokine secretion	C-C chemokine receptor type 2 antagonist, ALKBH5 inhibitor, arginase 1 agonist	(Wu Y. et al., 2023) (Liu et al., 2023) (Cai et al., 2019)

#### 3.1.3 Current consensus and controversies

Across multiple studies, consistent evidence shows that BCAAs catabolic enzymes are downregulated in failing hearts leading to BCAAs accumulation, that GLS1 upregulation is a conserved feature of activated myofibroblasts across fibrosis models, and that pharmacological GLS1 inhibition consistently reduces collagen deposition (Uddin et al., 2019) (Cui et al., 2019). However, key controversies remain: whether BCAAs accumulation directly drives fibrosis or merely reflects mitochondrial dysfunction, whether Gln dependency is specific to myofibroblasts or shared with inflammatory macrophages, and whether the promising preclinical efficacy of GLS inhibitors will translate to cardiac diseases given their predominant oncology-focused development. These discrepancies likely stem from differences in experimental models, cell-type heterogeneity, and the use of pharmacological inhibitors versus genetic approaches, highlighting the need for cell-type-specific metabolic flux analysis and lineage-tracing models to resolve them.

### 3.2 Reprogramming of glucose metabolism

Glycolysis of normal cells is mainly enhanced under hypoxic conditions. However, in cardiac tissue with sufficient nutritional supply, myocardial fibroblasts still tend to increase the glycolysis rate. This is because, although the efficiency of oxidation and phosphorylation under aerobic conditions is higher, glycolysis produces ATP faster than glucose oxidation in mitochondria (Wang and Patti, 2023). Moreover, it has been suggested that glycolysis may be spatially more efficient in generating ATP compared to glucose oxidation, given that cells have limited physical volume (Ritterhoff et al., 2020). Additionally, intermediate metabolites produced by glycolysis, such as glucose-6-phosphate and pyruvate, play a crucial role in the synthesis of nucleotides, lipids, and amino acids, which are essential for fibroblast proliferation and the repair of damaged cardiomyocytes (Prieto et al., 2021) (Flam and Arany, 2023). Under stress, damaged cardiomyocytes may experience impaired mitochondrial function due to ischemia-reperfusion or oxidative stress, compelling the cells to rely on glycolysis for energy production even when sufficient oxygen is available. Therefore, pathological changes in damaged cardiomyocytes are often accompanied by metabolic reprogramming of the heart, leading to increased dependence on glycolysis (Zhou et al., 2022). In the pathological process of cardiovascular diseases, fibroblast-to-myofibroblast transformation (FMT) plays a pivotal role in driving the cardiac fibrotic response (Ma et al., 2023). Using a mouse model of MI and related experiments, it has been demonstrated that interleukin-22 can reduce the expression of glycolysis-related enzymes (such as hexokinase 2 (HK2), phosphofructokinase-2/fructose-2,6-bisphosphatase 3 (PFKFB3), and pyruvate kinase isoform M2 (PKM2)), as well as lactic acid production, by decreasing the activity of these enzymes in cardiac fibroblasts, thereby inhibiting cardiac fibrosis and the FMT process following MI (Che et al., 2021). In contrast, angiotensin II promotes cardiac fibrosis by upregulating the expression of glycolysis-related genes, such as lactate dehydrogenase A (LDHA) (Hailiwu et al., 2023) and PKM2 (Zhang X. et al., 2021). Research has shown that interleukin-22 mitigates cardiac fibrosis by regulating metabolic reprogramming, particularly by down-regulating aerobic glycolysis levels in cardiac fibroblasts through the activation of the JNK/PKM2 pathway (Liu F. et al., 2024) (Chen et al., 2020). After MI, genes associated with myocardial fibroblast metabolism undergo changes, with the glycolytic pathway being the most significantly affected. Specifically, this manifests as upregulation of key glycolytic enzymes, increased glucose uptake and enhanced lactate production (Chen et al., 2023). It has been demonstrated that neonatal rat cardiac fibroblasts treated with (TGF-β1, 10 ng/ml) and induced with 3-(3-pyridinyl)-1-(4-pyridinyl)-2-propen-1-one (3PO) (20 μM), with or without a PFKFB3 inhibitor, for 24 hours. Analysis of the subsequent experimental results revealed that PFKFB3 was upregulated in TGF-β1-treated cells, while 3PO downregulated its expression. Concurrently, *in vivo* experiments confirmed this finding: in mice undergoing MI surgery, administration of 3PO or DMSO every other day resulted in 3PO significantly reducing PFKFB3 expression post-infarction and attenuating excessive fibrosis (Yang et al., 2023). Pharmacological inhibition of PFKFB3 alleviates the TGF-β1-mediated pro-fibrotic phenotype, thereby mitigating cardiac fibrosis and protecting cardiac function (Wang et al., 2023) (Liu C. et al., 2024). This suggests that glucose metabolism reprogramming is regulated by multiple factors during cardiac fibrosis and that complex interactions exist between these factors.

Across multiple studies, consistent evidence shows that glycolysis is significantly upregulated in cardiac fibroblasts following MI or TGF-β stimulation; PFKFB3 and HK2 are key drivers of this metabolic shift; and pharmacological or genetic inhibition of these glycolytic enzymes attenuates fibroblast activation and collagen deposition (Wang et al., 2023). However, it remains controversial whether this metabolic shift is a driver or merely a permissive adaptation for fibrosis. For instance, lactate accumulation has been linked to histone lactylation and fibroblast activation, but whether lactate directly triggers fibrosis in the absence of TGF-β remains untested. Furthermore, most glycolytic inhibitor studies (such as 2-DG and 3PO) have been conducted in acute injury models, and their effects in chronic, established fibrosis are unknown. Additionally, fibroblast-specific deletion of key glycolytic enzymes would help establish causality.

### 3.3 Lipid metabolism reprogramming

#### 3.3.1 Physiological functions of lipids and aberrant lipid metabolism

In addition to being essential components of cell and organelle membranes, lipids (such as triglycerides (TG), phospholipids, and sterols) serve as second messengers for intracellular signaling (Zeng et al., 2024). Additionally, lipids are the most significant bioenergetic substrates, providing substantial energy for the continuous pumping of blood in the heart, thereby directly influencing cardiac cellular homeostasis and function (Glatz et al., 2024). However, a few recent studies show that abnormal lipid metabolism has the effect of promoting fibrosis and participates in the occurrence and development of cardiac fibrosis (Schulze et al., 2016). As a high energy consuming organ of the human body, the energy metabolism of the heart mainly relies on the beta-oxidation pathway of fatty acids to drive the energy supply of OXPHOS (Rodolico et al., 2023). Studies have shown that in the process of cardiovascular disease, the main energy substrate of the heart changes, and the energy supply pattern changes from FAO to glucose utilization, resulting in fatty acid intake-oxidation imbalance. This metabolic disturbance may trigger pathological lipid deposition in cardiomyocytes, characterized by the abnormal accumulation of TG, diacylglycerol (DAG), ceramides, and cholesterol metabolites (Da Dalt et al., 2023). Among them, ceramide and DAG, as key cardiac toxic molecules, may eventually drive cardiac fibrosis and accelerate myocardial cell apoptosis by mediating insulin resistance and mitochondrial function damage (Actis Dato et al., 2024) (Hadas et al., 2020).

#### 3.3.2 The relationship between CPT1 and cardiac fibrosis

Under the background of cardiac fibrosis, myocardial cells and activated fibroblasts (myofibroblasts) show changes in metabolic characteristics. For example, the activity of acetyl coenzyme A carboxylase (ACC) in these cells is enhanced, resulting in an increase in the level of propionic coenzyme A, thus inhibiting the activity of CPT1. CPT1, the rate-limiting enzyme of mitochondrial fatty acid oxidation, regulates the β-oxidation rate by converting long-chain acyl-CoA to acylcarnitine, thereby facilitating its mitochondrial uptake (Lin et al., 2024). A transverse aortic constriction (TAC)-induced pressure overload assay in heterozygous CPT1b knockout (KO) mice (CPT1b+/−) showed that the hearts of CPT1b+/− mice exhibited abnormal mitochondrial function and abnormal accumulation of myocardial lipids, with elevated levels of TG and ceramides, which resulted in increased apoptosis and pronounced fibrosis in cardiomyocytes (He et al., 2012).

#### 3.3.3 Role of endoplasmic reticulum stress and lipid synthesis in cardiac fibrosis

In cardiomyocytes under pathological conditions such as ischemia, hypoxia, or oxidative stress, unfolded or misfolded proteins accumulate in the endoplasmic reticulum (ER), triggering ER stress (ERS) (Horn and Tacke, 2024). Persistent ERS can promote cardiac fibrosis by activating downstream signaling pathways. ERS induces the unfolded protein response via IRE1α, PERK, and ATF6 (Lebeaupin et al., 2018). It also promotes the maturation and nuclear translocation of sterol regulatory element−binding proteins (SREBPs) through PERK or IRE1α, upregulating lipid synthesis−related genes and leading to intracellular lipid deposition (Dong et al., 2024). The SREBP family contains three isoforms: SREBP−1a regulates fatty acid and cholesterol biosynthesis; SREBP−1c controls fatty acid and triacylglycerol synthesis; SREBP−2 governs cholesterol synthesis (Kawamura et al., 2022). SREBPs also regulate ACC, fatty acid synthase (FASN), and stearoyl−CoA desaturase 1 (SCD1) (Nakagawa et al., 2018). In a high−sugar/high−fat feeding study, SREBP−1c knockout mice showed significantly reduced fibrosis markers (COL−1, COL−3, α−SMA) compared to wild−type controls, confirming the link between lipid synthesis and cardiac fibrosis (Lee et al., 2024).

#### 3.3.4 Effects of lipid overload and metabolic abnormalities on cardiac fibrosis

Intracellular lipid overload in cardiomyocytes results in mitochondrial dysfunction and an increase in reactive oxygen species (ROS), which can lead to apoptosis or necrosis and the subsequent release of pro-fibrotic factors such as TGF-β, thereby activating cardiac fibroblasts (Ryu et al., 2026) (Ross and Benjamin, 2025). In addition, abnormal lipid metabolism will further aggravate the inflammatory microenvironment and promote the transformation of fibroblasts into muscle fibroblasts (Chen et al., 2026a). This process involves cross-regulation of multiple pathways, indicating that combined intervention on lipid metabolism may be a new direction for the treatment of cardiac fibrosis Crosstalk and integration between metabolic pathways.

Across multiple studies, consistent evidence shows that FAO is impaired in failing hearts while lipid accumulation is increased, that lipotoxicity mediated by ceramides and DAG promotes fibroblast activation and cardiomyocyte apoptosis, and that targeting lipid metabolic enzymes such as ACC1 or CD36 ameliorates cardiac fibrosis (Zhang Y. et al., 2021). Although lipid accumulation (particularly ceramides and DAG) is significantly associated with fibrosis, it remains controversial whether lipid metabolism abnormalities are a driving factor of fibrosis or merely a concomitant phenomenon of heart failure. Most intervention studies have employed global gene knockout approaches (such as CPT1b and SREBP-1c), which cannot distinguish the relative contributions of cardiomyocytes, fibroblasts, and macrophages. Furthermore, whether enhanced FAO might paradoxically promote fibrosis due to increased ROS requires investigation through cell-type-specific metabolic regulation studies.

### 3.4 Crosstalk and integration between metabolic pathways

Traditional metabolic studies often segregate glucose, lipid, and amino acid pathways. However, during the process of cardiac fibrosis, glucose, lipid, and amino acid metabolism do not operate in isolation; rather, they form a complex network of interactions through the sharing of metabolic intermediates, cofactors, and regulatory signals. Understanding this crosstalk is essential for developing effective therapeutic strategies that target the fibrotic process.

The interface between glycolysis and lipid metabolism is mediated by key metabolites. Pyruvate-derived oxaloacetate combines with acetyl-CoA to form citrate, which is cleaved by ATP-citrate lyase (ACLY) to generate acetyl-CoA for *de novo* fatty acid and cholesterol synthesis. In activated cardiac fibroblasts, ACLY inhibition suppresses TGF−β−induced fibroblast activation and fibrosis (Kuwahara et al., 2025) (Lazaropoulos et al., 2024). Meanwhile, the pentose phosphate pathway, a glycolytic branch, provides NADPH and pentoses to support reductive biosynthesis and nucleotide synthesis, promoting fibroblast proliferation in fibrotic hearts (Chen Z. et al., 2025) (Wang et al., 2022). Thus, glycolysis supplies both energy and carbon skeletons for pro−fibrotic lipid synthesis.

A direct link between glucose and amino acid metabolism occurs via glycine synthesis from the glycolytic intermediate 3−phosphoglycerate (3−PG). Glycine is a major component of collagen. Under TGF−β stimulation, activated fibroblasts upregulate the *de novo* serine/glycine synthesis pathway (via phosphoglycerate dehydrogenase (PHGDH) and serine hydroxymethyltransferase); inhibiting this pathway reduces collagen production (Satyanarayana et al., 2021) (Nigdelioglu et al., 2016). Although direct evidence in cardiac fibroblasts is limited, enhancing PHGDH−mediated serine synthesis in a dilated cardiomyopathy mouse model reduces cardiac fibrosis (Kay et al., 2026), suggesting a functional role and a potential therapeutic target in cardiac fibrosis.

Lipid and amino acid metabolism intersect at multiple levels. Acetyl-CoA, derived from fatty acid β−oxidation and BCAA catabolism, serves as a substrate for histone acetylation (He et al., 2023). Glutamine (Gln) metabolism is particularly prominent: α−KG from Gln breakdown acts as a cofactor for prolyl hydroxylase, stabilizing HIF−1α and driving glycolysis and lipid synthesis (Ge et al., 2018). During cardiac fibroblast−to−myofibroblast transition, Gln catabolism increases, enhancing α−KG bioavailability and promoting myofibroblast differentiation via chromatin remodeling. GLS1 inhibition (pharmacological or genetic) blocks myofibroblast transformation in human and mouse cardiac fibroblasts, as shown by decreased α−SMA, collagen gel contraction, and collagen synthesis. Notably, fibroblasts from failing human hearts also show suppressed myofibroblast phenotypes upon Gln degradation inhibition, indicating a functionally significant role in human cardiac fibrosis (Gibb et al., 2022a). In summary, lipid and amino acid metabolism interact through acetyl−CoA−mediated epigenetic regulation and Gln catabolism−driven myofibroblast differentiation, jointly promoting cardiac fibrosis.

**Figure 1:**
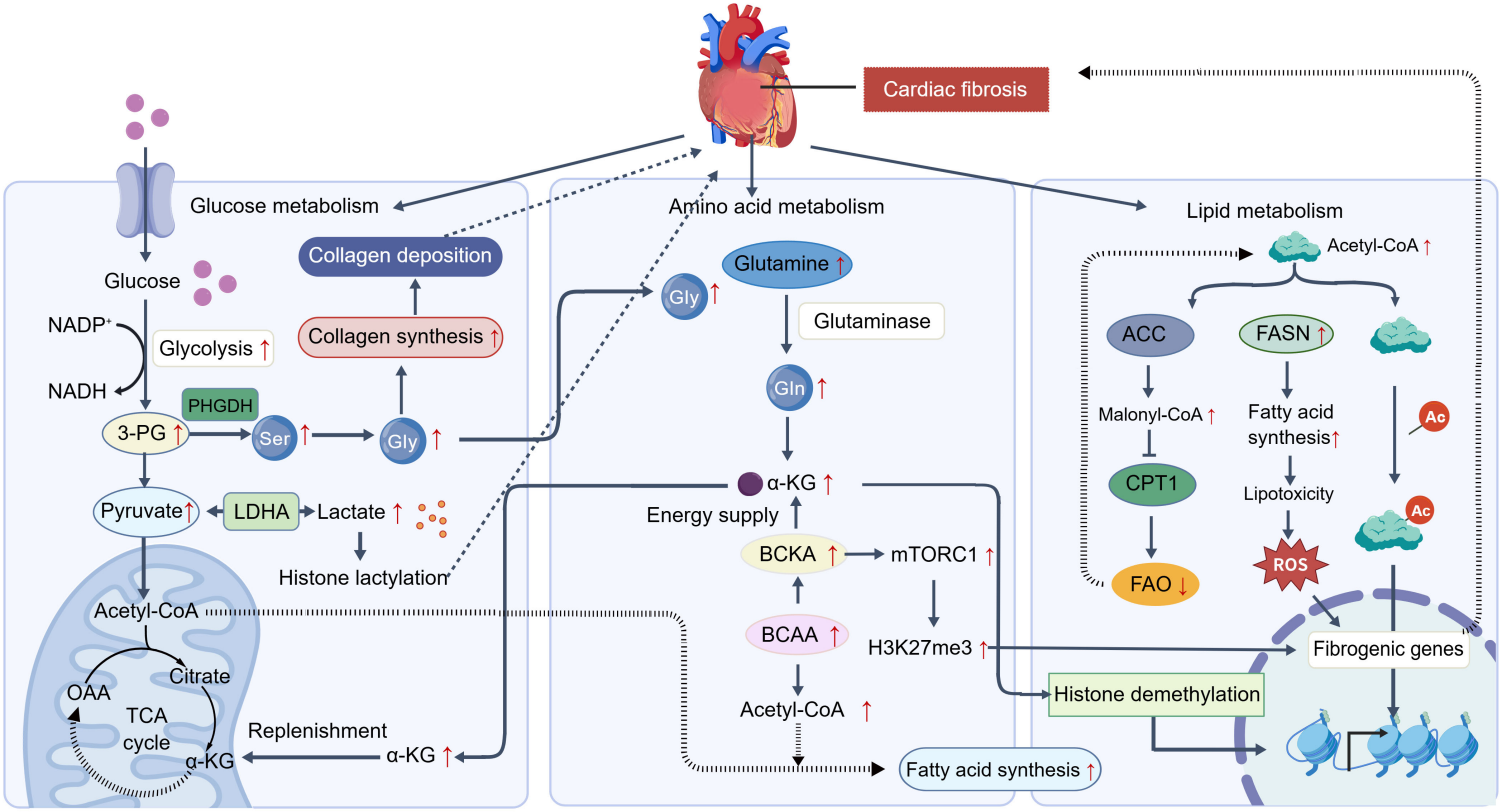
Crosstalk of metabolic reprogramming in cardiac fibrosis. Overview of crosstalk of metabolic reprogramming including glucose metabolism, lipid metabolism, and amino acid metabolism, as well as their crosstalk through shared intermediates (such as acetyl-CoA and α-KG) and epigenetic regulation in cardiac fibrosis.↑, increase/activation; ↓, decrease/inhibition. Ac, acetylation; Ser, serine; Gly, glycine; OAA, oxaloacetate. Created with BioGDP.com.

The crosstalk between glucose, lipid, and amino acid metabolism in cardiac fibrosis is summarized in **Figure 1** (Jiang et al., 2025).

Recent studies indicate that glucose, lipid, and amino acid metabolic reprogramming converge on ferroptosis—an iron-dependent, lipid peroxidation-driven cell death implicated in cardiac fibrosis. In hypoxic or highly glycolytic cardiomyocytes, lactate accumulation causes microenvironmental acidification, which impairs iron transporter function, leading to intracellular iron overload. Free iron then catalyzes ROS production via the Fenton reaction, triggering lipid peroxidation and ferroptosis (Jiang et al., 2021). Ferroptotic cardiomyocytes release damage-associated molecular patterns (DAMPs) that activate neighboring fibroblasts, promoting their transformation into myofibroblasts and exacerbating ECM deposition. Moreover, acidic conditions may suppress glutathione (GSH) synthesis, weakening the antioxidant capacity of GPX4 (which depends on GSH and targets lipid peroxides). Notably, GSH synthesis requires glutamine metabolism, linking amino acid and lipid pathways (Rodríguez-Graciani et al., 2022). In cardiac fibroblasts, GPX4 downregulation correlates with increased collagen deposition, and GPX4 inhibition worsens fibrosis (Cheng et al., 2024). Thus, ferroptosis serves as a common executioner: lactate-driven acidification (glucose metabolism) initiates iron overload and lipid peroxidation (lipid metabolism), while glutamine-dependent GSH/GPX4 (amino acid metabolism) modulates the process. This triple convergence drives cardiac fibrosis progression.

## 4 The mechanism of metabolism reprogramming to promote cardiac fibrosis

### 4.1 Fatty acid oxidation inhibition and glycolysis dominate

In the fibrotic heart, metabolic reprogramming is primarily governed by glycolysis, leading to impaired OXPHOS and reduced ATP production efficiency. This inadequacy fails to meet the requirements for sustained vigorous myocardial contraction and normal fibroblast metabolism, ultimately resulting in decreased contractility (Rhana et al., 2024). The dominance of glycolysis results in diminished activity of the mitochondrial respiratory chain complex and accumulation of ROS, further disrupting mitochondrial membrane potential and exacerbating the imbalance in energy metabolism (Harachi et al., 2020).

### 4.2 The role of metabolite accumulation in cardiac fibrosis

Metabolite accumulation is a key feature of cardiac fibrosis. Certain metabolites promote the progression of fibrosis through oxidative stress, inflammation, and epigenetic reprogramming, while others may exert protective effects. This section discusses the evidence base for both pro-fibrotic and anti-fibrotic metabolites.

#### 4.2.1 Lactic acid-driven fibrosis vicious circle

*In vivo* experiments have demonstrated that peritoneal injection of the glycolysis inhibitor 2-deoxy-D-glucose (2-DG) in mice with myocardial infarction (MI) reduces serum and cardiac lactate levels elevated by MI. Statistical analysis and Masson’s trichrome staining revealed that 2-DG administration attenuated cardiac fibrosis, suggesting that enhanced glycolysis and subsequent lactate accumulation contribute to cardiac fibrosis and cardiac dysfunction in a causal manner (Fan et al., 2023). During myocardial ischemia, hypoxia, or heart failure, cardiomyocytes rely on anaerobic glycolysis to produce ATP, resulting in the generation of substantial amounts of lactate. The accumulation of lactate leads to a decrease in intracellular pH, inducing acidosis and the activation of acid-sensitive ion channels and sodium-potassium ion exchangers. This process results in an increased inward flow of sodium ions and calcium ion overload, which further damages cardiomyocytes (Wu et al., 2022). Local acidosis induced by lactate production enhances TGF-β activity, promotes the synthesis and deposition of Collagen type I (COLI) and Collagen type III (COLIII) and further stimulates lactate production through hypoxia-inducible factor-1alpha signaling (Zhu et al., 2024), thereby exacerbating cardiac fibrosis. Studies have shown that lactate promotes collagen deposition through histone lactylation modifications, facilitating its nuclear translocation and activating the Endothelial-to-Mesenchymal Transition (EndoMT) and TGF-β/Smad family member 2 (Smad2) signaling pathways (Ayala et al., 2014).

It is worth noting that inhibiting lactate production *in vivo* can directly reduce cardiac fibrosis, and this effect is independent of its impact on mortality. *In vitro* experiments further confirm this direct effect: by inhibiting glycolysis and lactate production, 2-DG can directly block the activation of TGF-β-activated cardiac fibroblasts. Together, these results indicate that lactate accumulation is not merely a metabolic byproduct, but a pathogenic factor that directly drives the progression of fibrosis (Chen et al., 2021).

#### 4.2.2 Lipid peroxidation and inflammation amplification

Lipid peroxides undergo degradation, producing various toxic lipid aldehydes, including alkanals, allyl aldehydes, hydroxynonenals, and enedienes. Among these, 4-hydroxynonenal (4-HNE) and malondialdehyde (MDA) are recognized as the most prevalent toxic by-products of lipid peroxidation. These compounds can cross-link with amino groups in cell membrane phospholipids, such as forming MDA-protein adducts, thereby damaging cell membranes, mitochondria, and other organelles, and initiating apoptotic or inflammatory responses (Zhang et al., 2023). As the most abundant and toxic aldehyde derived from lipid peroxidation, 4-HNE is closely linked to the progression of several diseases, including cardiovascular diseases and cancer (Sonowal and Ramana, 2019). It can bind to components of the mitochondrial respiratory chain complex, inhibit ATP synthesis, and promote an increase in ROS.

NF-κB is a heterodimeric transcription factor. 4-HNE can exacerbate inflammatory damage by regulating the NF-κB signaling pathway (which manifests as the promotion of nuclear translocation in most vascular cells), thereby increasing the release of inflammatory cytokines such as tumor necrosis factor-alpha, interleukin-6 (IL-6), IL-1 and Monocyte chemoattractant protein-1 (Gianazza et al., 2019). Furthermore, ROS and inflammatory factors further activate TGF-β, promoting fibroblast proliferation and collagen synthesis, and inducing cardiomyocyte death and interstitial fibrosis. During oxidative stress, increased ROS production triggers a cascade of lipid peroxidation and degradation, leading to the formation of lipid peroxides. This creates a vicious cycle that further promotes fibrosis (Ji et al., 2022).

Notably, the removal of lipid peroxides can directly influence NF-κB activation and the fibrotic process. In cardiac microvascular endothelial cells, activation of the 4-HNE detoxifying enzyme ALDH2 restores VEGFR2 expression and angiogenic function that had been inhibited by 4-HNE, while further activation of NF-κB enhances this protective effect of ALDH2 (Roy et al., 2026). In the post-myocardial infarction heart, overexpression of glutathione S-transferase M1 can alleviate cardiac fibrosis by inhibiting lipid peroxidation and ferrocytosis (Chen KJ. et al., 2025). These studies indicate that intervening in the production of lipid peroxidation products or accelerating their clearance can reduce fibrosis, suggesting that 4-HNE and MDA are not merely markers of oxidative stress, but key pathogenic mediators directly involved in the progression of fibrosis.

#### 4.2.3 Abnormality of amino acid metabolism

Disruptions in amino acid metabolism can lead to abnormal accumulation of toxic byproducts such as ammonia and branched-chain ketone acids (BCKAs), disrupting the stability of the cellular microenvironment and interfering with the metabolic flux of α-ketoglutarate (α-KG) in the TCA cycle. This, in turn, inhibits ATP production and exacerbates energy metabolism disorders. These abnormalities further disrupt cellular metabolic balance and affect cellular signaling, ultimately promoting myocardial injury and fibrosis (Wen et al., 2025) (Sun et al., 2016). However, most current studies in this field have only revealed a correlation between amino acid metabolic abnormalities and cardiac fibrosis; rigorous genetic and cell-specific evidence to confirm a causal relationship is still lacking.

Taking GLS1 as an example, recent evidence including fibroblast-specific GLS1 knockout and CB-839 administration has confirmed in pressure overload heart failure models that it can reverse established cardiac fibrosis, suggesting that glutamine metabolism may be an upstream driver. However, existing evidence still has limitations. Systemic administration makes it difficult to exclude off-target effects, data on primary cardiac fibroblasts are limited, and other amino acid pathways such as branched-chain amino acids and tryptophan still lack causal validation. Therefore, the causal relationship between amino acid metabolism and cardiac fibrosis requires further confirmation through more cell-specific and temporally controlled genetic intervention studies (Gibb et al., 2022b).

#### 4.2.4 Ketone body metabolism: a complex role in cardiac fibrosis

In addition to the classic remodeling of carbohydrate, lipid, and amino acid metabolism, recent studies have revealed that ketone body metabolism also undergoes significant reprogramming during heart failure and has been shown to act as a regulator of cardiac fibrosis.

##### 4.2.4.1 Ketone body formation and metabolism

Ketone bodies include acetoacetate, β-hydroxybutyrate (β-OHB), and acetone. Under conditions of insufficient glucose supply, such as fasting, starvation, or prolonged exercise, fatty acid β-oxidation in liver mitochondria produces large amounts of acetyl-CoA, which is converted into ketone bodies via HMG-CoA synthase and HMG-CoA lyase. In addition, acetyl-CoA generated from the breakdown of ketogenic amino acids (leucine and lysine) can also produce ketone bodies. This dual source places ketone body metabolism at the intersection of lipid and amino acid metabolism (Abdul Kadir et al., 2020).

##### 4.2.4.2 Ketone body metabolism in the heart

In a healthy heart, the contribution of ketone body oxidation to ATP production is minimal. After entering cardiomyocytes via the monocarboxylate transporters MCT1/2, ketone bodies are primarily converted into acetoacetate in the mitochondria by β-OHB catalyzed by BDH1, and then further converted by succinyl-CoA: 3-keto-CoA transferase (SCOT), which accepts CoA from succinyl-CoA to form acetyl-CoA, which is subsequently cleaved into two molecules of acetyl-CoA and enters the TCA cycle. SCOT is the key rate-limiting enzyme in myocardial ketone body oxidation, mediating the transfer of the CoA group from succinyl-CoA to acetoacetate (Yurista et al., 2021). However, in the failing heart, ketone body oxidation is significantly upregulated. Aubert et al. (2016) were the first to systematically demonstrate in a mouse model that, compared to non-failing hearts, the flux of ketone body oxidation in failing hearts increased by approximately 2–3-fold, accompanied by upregulation of BDH1 and SCOT protein expression (Aubert et al., 2016). Human end-stage heart failure samples similarly confirmed enhanced ketone body utilization and elevated SCOT expression, and this metabolic remodeling was independent of diabetic status (Bedi et al., 2016). Both clinical and basic research have found that the degree of upregulation of myocardial ketone body oxidation is positively correlated with the severity of heart failure (de Koning et al., 2021), suggesting that it represents an endogenous compensatory adaptation: when heart failure is accompanied by impaired FAO and reduced glucose oxidation efficiency, upregulated ketone body oxidation can maintain mitochondrial OXPHOS function, compensating for energy metabolism defects and reducing the myocardium’s overreliance on glucose.

**Figure 2:**
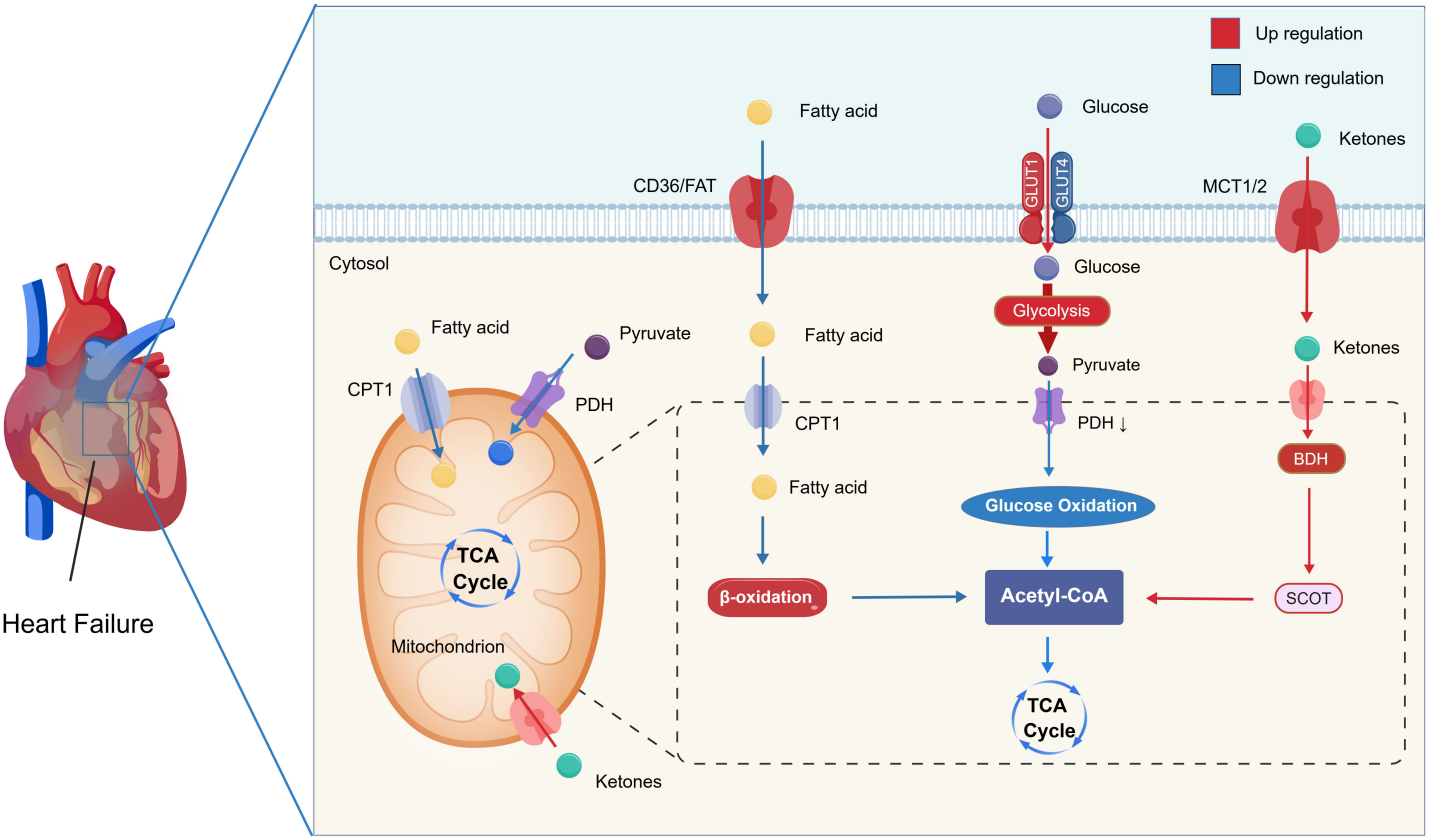
Schematic diagram of cardiac metabolic reprogramming in heart failure. Blue arrows indicate downregulation; red arrows indicate upregulation. During the progression of heart failure, fatty acid β−oxidation and glucose oxidation show a decreasing trend, while ketone body oxidation is compensatorily increased. MCT, monocarboxylate transporter; CD36/FAT, fatty acid translocase; PDH, pyruvate dehydrogenase; SCOT, succinyl−CoA:3−ketoacid CoA transferase. Created with BioGDP.com.

It should be noted that the changes in FAO in heart failure are stage−dependent (Stanley et al., 2005). In the early stage of heart failure, both FAO and glucose oxidation show a decreasing trend (Fukushima and Lopaschuk, 2016), while ketone body oxidation is compensatorily increased (**Figure 2**) (Aubert et al., 2016; Jiang et al., 2025). As the disease progresses to the late stage, FAO is further impaired due to mitochondrial dysfunction, and glucose oxidation becomes limited because of uncoupling and worsening mitochondrial dysfunction (Sun et al., 2024). However, conflicting results also exist, with some heart failure patients showing no decline in FAO. These discrepancies likely depend on the heart failure subtype, disease severity, and the characteristics of the study populations.

Currently, the specific molecular mechanisms driving the upregulation of myocardial ketone body oxidation in heart failure have not been fully elucidated. Existing studies suggest that the activation of transcription factors such as PPARα participates in regulating the expression and functional remodeling of ketone body metabolism (Kersten, 2014; Grabacka et al., 2016). However, the upstream pathological triggers and the upstream and downstream regulatory networks still require further analysis.

##### 4.2.4.3 Anti-fibrotic mechanisms and experimental evidence of ketone body metabolism

In addition to serving as an energy substrate, β-OHB—the predominant form of ketone bodies in circulation—can also exert independent anti-fibrotic effects on the heart through epigenetic regulation and signaling pathways. The most well-established mechanism currently is the competitive inhibition of class I histone deacetylases (HDACs). β-OHB competitively binds to HDAC substrates, leading to increased histone acetylation levels in the promoter regions of antioxidant genes (such as FOXO3a and MT2). This epigenetic regulation reduces oxidative stress levels in cardiac fibroblasts, thereby inhibiting TGF-β1-induced myofibroblast transformation (Shimazu et al., 2013) (Lecoutre et al., 2022).

In a rat model of diabetic cardiomyopathy (DCM), intraperitoneal administration of β-OHB significantly improved cardiac function and reduced cardiac fibrosis. Mechanistic studies indicate that β-OHB exerts its antifibrotic effects by regulating macrophage polarization: in the myocardium of DCM rats, the proportion of pro-inflammatory M1 macrophages is elevated, while that of reparative M2 macrophages is reduced; this dysregulated inflammatory microenvironment further drives fibroblast activation and collagen deposition, whereas β-OHB treatment reduces M1 and increases M2 macrophages. Co-culture experiments using neonatal rat cardiac fibroblasts and peritoneal macrophages further confirmed that under high-glucose conditions, M1 macrophages increased while M2 macrophages decreased; however, β-OHB treatment reversed this trend while simultaneously reducing collagen expression in fibroblasts. These findings suggest that β-OHB reduces collagen secretion in cardiac fibroblasts by promoting M2 macrophage polarization (He et al., 2025).

Furthermore, in a mouse model of heart failure with preserved ejection fraction (HFpEF), supplementation with β-OHB increased myocardial β-OHB levels, improved myocardial mitochondrial function, reduced collagen synthesis, and downregulated the expression of genes associated with pre-fibrotic signaling (such as α-SMA and Timp1). The study further revealed that excessive mitochondrial acetylation accelerates NLRP3 inflammasome assembly, inducing myocardial inflammation and fibrosis, whereas β-OHB exerts a protective effect by targeting the mitochondrial-inflammation loop (Deng et al., 2021).

##### 4.2.4.4 Controversies and uncertainties

It should be noted that not all studies support the antifibrotic effects of ketone bodies. Studies have reported that long-term ketogenic diets (≥16 weeks) or β-OHB injections can induce cardiac fibrosis in rats, primarily by increasing β-OHB expression, activating SIRT7 transcription, and subsequently inhibiting mitochondrial biosynthesis, leading to cardiomyocyte apoptosis and fibrosis formation. This finding has also been observed in human atrial fibrillation cardiac tissue (Xu et al., 2021). Another study also found that a ketogenic diet exacerbates cardiac remodeling and fibrosis in spontaneously hypertensive rats (You et al., 2020). These conflicting results suggest that the effects of ketone bodies may be dose- and time-dependent: short-term ketosis may exert a protective effect, whereas long-term or higher levels of ketosis may produce the opposite effect. The dual “protective” and “damaging” effects of ketone bodies indicate strict dose- and time-dependence; however, there are currently no systematic studies evaluating the dose-response relationship between ketone body concentrations and the degree of fibrosis.

The causal relationship between abnormal ketone body metabolism and cardiac fibrosis remains to be clarified. Endogenous ketosis has been confirmed during heart failure, but it is currently unclear whether this ketosis actively inhibits fibrosis or merely reflects the severity of metabolic dysfunction. Finally, there have been no systematic studies on the response of fibroblasts derived from human hearts with heart failure to β-OHB and given the significant differences in metabolic characteristics between rodent and human hearts, directly extrapolating animal study results to clinical treatment has limitations.

### 4.3 Interactive activation of signal path

The regulation of the fibrotic process by metabolic reprogramming involves a complex, multi-pathway regulatory network. In addition to participating in energy metabolism and substance synthesis, metabolic enzymes and metabolites can also act as key signaling molecules to mediate the activation of downstream pathways, thereby driving the pathological progression of fibrosis.

Studies have confirmed that PKM2 regulates the JAK2/STAT3 signaling pathway and participates in the angiotensin II-mediated fibrotic damage process (Zhang X. et al., 2021); ACLY can upregulate NF-κB signaling pathway activity, induce the secretion of pro-fibrotic factors, and promote abnormal fibroblast proliferation (Chen et al., 2019). Targeted inhibition of NF-κB pathway activity, such as through pharmacological interventions with curcumin or vitamin D, can significantly reduce inflammation levels and decrease abnormal collagen deposition in tissues (Zeng et al., 2015) (Saleh et al., 2020).

Metabolites may also participate in fibrosis regulation through receptor-mediated mechanisms; among these, succinic acid can specifically bind to the SUCNR1 receptor, activate the MAPK signaling pathway, and promote the proliferation and activation of myofibroblasts (Shi Y. et al., 2025) (Wang et al., 2024). The aforementioned signaling pathways exhibit extensive cross-regulatory relationships with the classical TGF-β/Smad fibrosis pathway: NF-κB and TGF-β can form a positive feedback loop, mutually activating each other and continuously exacerbating the progression of fibrosis (Guo Q. et al., 2024); mTOR signaling can participate in the regulation of collagen metabolism by modulating autophagy levels, synergistically promoting the onset and development of fibrosis (Shi L. et al., 2025).

Importantly, these pathways form an interconnected network (such as TGF−β → mTORC1 → ROS → TGF−β). This network explains why metabolic reprogramming converges on a shared fibrotic program through energy imbalance, toxic metabolites, and multi−pathway interactions. Future research should integrate single−cell multi−omics and metabolic imaging to develop precise interventions targeting key metabolic nodes.

## 5 Pharmacological interventions

Metabolic reprogramming offers new therapeutic targets for cardiac fibrosis. Based on the degree of clinical translation of existing evidence, relevant interventions can be categorized into three tiers: clinically available anti-fibrotic metabolic drugs, preclinical metabolic modulators (validated in animal models), and emerging gene therapies and nutritional interventions.

### 5.1 Clinically available agents with emerging metabolic effects

The clinical safety of these drugs is well established. In addition to their existing indications, their newly discovered metabolic regulatory effects give them the potential for “repurposing” to treat cardiac fibrosis; much of the evidence supporting this comes from clinical studies or *post-hoc* analyses.

#### 5.1.1 Sodium-glucose cotransporter 2 inhibitors

Several clinically available agents, originally approved for other indications, have been recognized for their emerging metabolic effects that may benefit cardiac fibrosis.

SGLT2 inhibitor was originally used as a hypoglycemic acid. Interestingly, recent reports have revealed emerging evidence that SGLT2 inhibitors are used as cardiac fibrosis regulators. SGLT2 has shown great therapeutic potential in cardiac fibrosis such as diabetic cardiomyopathy, heart failure and ischemic cardiomyopathy. Based on preclinical and clinical research, we found that SGLT2 inhibitors mainly regulate signals such as TGF-β/Smad and mTOR, showing anti-fibrosis characteristics, mainly involving oxidative stress, autophagy and other processes. Although SGLT2 inhibitors have demonstrated significant cardiovascular benefits in large-scale clinical trials, their direct anti-fibrotic mechanisms in the heart require further investigation, and issues related to target specificity remain to be fully elucidated (Karakasis et al., 2025).

#### 5.1.2 Lipid-modifying drugs: fibrates and ezetimibe

As PPAR-α agonists, the core mechanism of fibrates is to activate PPAR-α to regulate lipid metabolism: they upregulate CD36 and fatty acid transport proteins to promote fatty acid uptake and increase acyl-CoA synthetase to provide substrates for β-oxidation. They upregulate Lipoprotein lipase and inhibit Apolipoprotein C-III (ApoC-III) to promote the hydrolysis of triglyceride-rich lipoproteins and enhance the synthesis of ApoA-I/A-II, thereby increasing high-density lipoprotein cholesterol (HDL-C) (Kim and Kim, 2020). Animal studies have shown that fenofibrate reduces myocardial lipid accumulation, improves mitochondrial function, and alleviates fibrosis, with these effects being PPARα-dependent (Park et al., 2024). Furthermore, it inhibits the NF-κB signaling pathway through a non-PPARα-dependent mechanism, reducing the expression of pro-inflammatory factors such as IL-6 and tumor necrosis factor-alpha, as well as cardiac remodeling mediators such as matrixmetalloproteinase-9 and connective tissue growth factor, thereby improving cardiac function (Cevey et al., 2017). Clinical evidence also demonstrates that treatment with fibrates reduces the risk of major adverse cardiovascular outcomes, a process associated with low-density lipoprotein cholesterol (LDL-C) reduction. Notably, the clinical benefits are greater when fibrates are used in combination with statins. However, frequent use of statins may reduce the efficacy of fibrates (Kim et al., 2024).

In hypercholesterolemia, cholesterol oxidation products activate inflammatory pathways, promoting the transformation of cardiomyocytes into myofibroblasts and collagen deposition. The lipotoxic environment also promotes macrophage infiltration and the release of interferon gamma, IL-6, and other pro-fibrotic factors (Mostafa Arabi et al., 2022). In a mouse model of intestinal SREBP-2 deficiency, ezetimibe activated SREBP-2 proteolysis, reduced endogenous cholesterol synthesis, and improved lipid metabolism, thereby alleviating fibrosis (Rong et al., 2017). Clinical evidence has also demonstrated that ezetimibe specifically inhibits Niemann-Pick C1-like 1, blocks cholesterol absorption (by 67%), reduces LDL-C by approximately 15–20%, increases HDL-C, and is effective for hypercholesterolemia. Ezetimibe primarily exerts its effects by improving LDL-C levels in patients at high risk for cardiovascular disease or those intolerant to statins. This class of drugs is recommended for use in combination with statins or for patients’ intolerant to statins (Mostafa Arabi et al., 2022) (Chilbert et al., 2022).

### 5.2 Preclinical metabolic modulators with anti-fibrotic potential

These drugs have demonstrated anti-fibrotic efficacy in animal models; however, evidence supporting their clinical translation is currently insufficient, and further evaluation of their safety and efficacy is required.

#### 5.2.1 Inhibitors of key enzymes in glycolysis

Inhibitors of key enzymes of glucose metabolism treat cardiac fibrosis by intervening in the glycolytic pathway. HK2 inhibitors (2-DG, clonidine) improve fibrosis by inhibiting glucose phosphorylation and reducing glycolytic overactivation, lactate accumulation, and pro-fibrotic signals (such as TGF-β), but there are side-effects, such as hypoglycemia, cardiotoxicity and the risk that inhibition of HK2 alone may trigger mitochondrial dysfunction; PFKFB3 inhibitors (such as 3PO) inhibit glycolytic flux and EndoMT by lowering fructose-2,6-bisphosphate levels and effectively attenuate collagen deposition and fibrosis, but their non-specificity limits their clinical application; PKM2 activators (such as TEPP-46) enhance enzyme activity by facilitating the formation of PKM2 tetramers, reduce the accumulation of glycolytic intermediates, and promote OXPHOS, thereby improving cardiomyocyte death and fibrosis, but specificity for PKM1 needs to be ensured. Future research and development should focus on improving targeting and reducing toxicity of inhibitors of key enzymes of glucose metabolism (Yin et al., 2019; Luo et al., 2020; Chen et al., 2021; Gao et al., 2022; Zeng et al., 2022; Guo et al., 2023; Yang et al., 2023; Zhang et al., 2025) (**Table 2**).

**Table 2:** The mechanism and application of glycometabolic enzyme inhibitors in cardiac fibrosis.

Target	Inhibitor/modulator	Mechanism	Effect	Limitations	References
HK2	2-DG	Competes with glucose at HK2, reducing lactate accumulation, intracellular and TGF-β signaling.	In the cardiac fibrosis model, 2-DG-treated mice showed a smaller collagen volume fraction and a weaker fluorescence signal of α-SMA than PBS.	Hypoglycemia, dizziness, and cardiac arrest have limited therapeutic benefit when 2-DG is offered as a single drug.	(Chen et al., 2021) (Luo et al., 2020)
	Lonidamine	Competitively inhibits HK2 and suppresses glycolytic flux.	Inhibits FMT and collagen deposition in fibroblasts.	Preclinical evidence mainly from pulmonary fibrosis, insufficient cardiac validation and potential off-target effects.	(Yin et al., 2019)
PFKFB3	3PO	Inhibits PFKFB3, reduces fructose 2,6-bisphosphate levels, restrains glycolytic flux, and suppresses the TGF-β1/Smad2/3 signaling pathway	Significantly reduced collagen density and fibrotic area in the infarcted region and decreased the expression of COLI, COLIII, and fibronectin.	Limited to animal experiments, lacking clinical research and sufficient safety evaluation	(Yang et al., 2023) (Zeng et al., 2022)
PKM2	TEPP-46	Promotes PKM2 tetramerization and OXPHOS in cardiomyocytes.	Promotes OXPHOS in cardiomyocytes, reducing cell death and cardiac fibrosis.	PKM2 and PKM1 are highly homologous but functionally distinct; it is important that regulators do not interfere with PKM1.	(Gao et al., 2022) (Guo et al., 2023) (Zhang et al., 2025)

#### 5.2.2 Lipid metabolism targets: acetyl-CoA carboxylase 2 inhibitors

In addition to approved lipid-lowering drugs (fibrates, ezetimibe), preclinical drugs targeting lipid metabolism pathways have also demonstrated potential for preventing cardiac fibrosis in animal models. Taking ACC2 inhibitors as an example, ACC2 inhibits the transport of fatty acids to mitochondria by generating malonyl-CoA; inhibiting ACC2 reverses this inhibitory effect, enhances fatty acid β-oxidation, and reduces lipid accumulation and lipotoxicity. In a heart failure model established using adipose tissue glycerol-3-phosphate lyase (ATGL) KO mice, Atgl/Acc2 double-KO mice exhibited significantly reduced myocardial triglyceride accumulation and fibrosis, as well as markedly improved cardiac function and survival rates. Following a single administration of the ACC2-selective inhibitor TLC-3595, propyl-CoA levels and triglyceride content in the myocardium of ATGL KO mice decreased in a dose-dependent manner; after continuous treatment, echocardiography revealed improved left ventricular ejection fraction and reduced cardiac remodeling and fibrosis. Metabolomic analysis further confirmed that TLC-3595 directly influences metabolic changes, including increased acylcarnitine levels in the myocardium, restoration of TCA cycle metabolites, and enhanced FAO. The above studies indicate that inhibiting ACC2 can effectively alleviate cardiac fibrosis. Currently, the ACC2 inhibitor (TLC-3595) has entered clinical development for type 2 diabetes, and its translational potential in cardiac fibrosis warrants further investigation (Choi et al., 2016) (Usui et al., 2025).

#### 5.2.3 Targets of amino acid metabolism

Targeting amino acid metabolic reprogramming is an emerging strategy for combating cardiac fibrosis. Currently, related drugs are in the preclinical research stage and primarily include GLS inhibitors and leucine sensor inhibitors.

GLS inhibitor (CB-839): GLS inhibitors, which target Gln metabolism, have exhibited anti-fibrotic effects in animal models of cardiac hypertrophy and heart failure. Excessive Gln metabolism exacerbates fibrosis, and the use of GLS inhibitors (such as CB-839) reduces glutamate accumulation and thus attenuates fibroblast activation. Results based on fibroblasts isolated directly from human failing hearts have shown that inhibition of Gln catabolism prevents TGF-β mediated differentiation and demonstrates great potential in reversing the enhanced activation phenotype. myofibroblasts were significantly reduced after CB-839 treatment (Gibb et al., 2022a).

Leucyl-tRNA synthetase 1 (LARS1) inhibitors: Leucine can activate the mTORC1 signaling pathway. However, excessive activation of mTORC1 may lead to cardiac fibroblast proliferation and collagen deposition (Yang et al., 2024) (Kim et al., 2021). LARS1 belongs to the aminoacyl-tRNA synthetase family; as a leucine sensor and metabolic regulator, it links intracellular leucine availability to protein synthesis. When leucine is abundant, it binds specifically to the leucine-binding pocket of LARS1, inducing a conformational change in LARS1 that activates its GTPase-activating protein function. This promotes the hydrolysis of GTP to GDP by RagD GTPase, leading to the formation of an active RagD-GDP/RagB-GTP heterodimer that activates mTOR (Sung et al., 2023) (Han et al., 2012). In addition, LARS1 can synergistically enhance the activation of mTORC1 via the Vps34-PLD1 pathway (Yoon et al., 2016). It has been demonstrated that inhibiting LARS1 attenuates TGF-β1-stimulated cellular lipid accumulation and epithelial-mesenchymal transition, while promoting lipophagy. Lipophagy is a specialized form of autophagy that plays a critical role in maintaining lipid homeostasis and cellular function. In a renal fibrosis model established using Lars1 heterozygous knockdown mice, LARS1 deficiency significantly suppressed the upregulation of COLI and α-SMA, thereby alleviating renal fibrosis. Additionally, in TGF-β1-treated renal tubular epithelial cells, LARS1 inhibition was found to reduce the TGF-β1-induced increase in ROS, as well as the TGF-β1-induced elevation of MDA and free fatty acids. Furthermore, LARS1 inhibited the TGF-β1-stimulated upregulation of fatty acid β-oxidation in these cells (Guo et al., 2025). Increased ROS reduced fatty acid β-oxidation capacity, and lipid deposition are pathological factors closely associated with cardiac fibrosis, and LARS1 inhibition can slow the progression of cardiac fibrosis. LARS1 small-molecule inhibitors, represented by BC-LI-0186, have demonstrated the potential to precisely block the leucine-mTORC1 signaling axis (Yoon et al., 2020) (Kim et al., 2017). Future research should explore the therapeutic value of such inhibitors in cardiac fibrosis models.

### 5.3 Emerging treatment strategies: gene therapy and nutritional interventions

#### 5.3.1 Gene therapy

With the rapid development of gene therapy and gene editing technologies, improving cardiomyocyte function and combating fibrosis by regulating metabolism-related gene expression has become a highly promising research direction (Grisorio et al., 2024) (Ishikawa et al., 2018).

##### 5.3.1.1 Enhancing mitochondrial oxidative capacity

Mitochondrial dysfunction is a hallmark of cardiac fibrosis, characterized by reduced FAO, impaired OXPHOS, and increased reliance on glycolysis. Enhancing mitochondrial oxidative capacity through gene therapy is a logical strategy for restoring metabolic homeostasis and slowing the progression of fibrosis.

Mitochondrial dysfunction is a major contributing factor to cardiac fibrosis; therefore, enhancing mitochondrial oxidative capacity is a key therapeutic strategy. Peroxisome proliferator-activated receptor gamma coactivator 1-alpha (PGC1α) is a major regulator of mitochondrial biogenesis and FAO (Guo M. et al., 2024) (Zhang et al., 2026). Clustered regularly interspaced short palindromic repeats (CRISPR) a-mediated transcriptional activation of PGC1α and mitochondrial transcription factor A, has been shown to enhance mitochondrial biogenesis and OXPHOS function (Shahannaz et al., 2026). Studies have shown that downregulation of SCD1 during the reprogramming of cardiac fibroblasts into induced cardiomyocytes significantly increases PGC1α expression. Subsequently, PGC1α collaborates with PPARβ to upregulate genes associated with FAO and mitochondrial biogenesis, thereby promoting cardiac transdifferentiation in induced cardiomyocytes and improving both their quality and quantity. These findings reveal the critical role of metabolic reprogramming in cardiac repair and suggest that PGC1α overexpression may be an effective strategy for improving myocardial energy metabolism (Jia et al., 2025).

##### 5.3.1.2 Correcting metabolic enzyme deficiencies

In the pathogenesis of dilated cardiomyopathy, impairment of the serine biosynthetic pathway plays a key role; this pathway is regulated by the rate-limiting enzyme PHGDH. A recent study employed an Adeno-associated virus 9 (AAV9) -mediated gene delivery strategy to upregulate PHGDH expression in a transgenic dilated cardiomyopathy mouse model. The results showed that fibrosis in the myocardial tissue of treated mice was significantly reduced, accompanied by decreased accumulation of pyruvate and lactate. Enhanced PHGDH maintained normal mitochondrial oxidative metabolism and redirected more carbon flux from glycolysis toward the serine biosynthetic pathway, thereby restoring serine levels in the heart and ultimately exerting a clear cardioprotective effect (Kay et al., 2026).

Overexpression of superoxide dismutase 2 and catalase has been shown to reduce myocardial injury (Chang et al., 2019). Delivery of antioxidant genes via AAV can enhance ROS scavenging capacity in cardiomyocytes and improve mitochondrial function (Biswal et al., 2017).

##### 5.3.1.3 Gene editing for pro-fibrotic metabolic regulator

Unlike approaches that enhance protective factors or correct metabolic enzyme deficiencies, strategies that directly edit pro-fibrotic metabolic regulatory genes aim to knock out or inhibit the metabolic enzymes that drive the fibrotic process using tools such as CRISPR-Cas9. These enzymes are abnormally overexpressed in activated fibroblasts, providing metabolic support for cell proliferation, collagen synthesis, and epigenetic reprogramming. Targeted intervention at these nodes holds promise for fundamentally disrupting the metabolic drive circuits of fibrosis.

For example, ACLY generates acetyl-CoA from citrate esters, providing substrates for fatty acid synthesis and epigenetic regulation. Recent studies indicate that TGFβ increases ACLY expression in cardiac fibroblasts; increased nuclear translocation of ACLY and its interaction with Smad transcription factors epigenetically drive fibrosis (Lazaropoulos et al., 2024). ACLY expression is upregulated in the hearts of mice treated with angiotensin II. AAV9-mediated silencing of the ACLY gene significantly reduced cardiac fibrosis, while inhibition of ACLY suppressed cardiac fibroblast proliferation and fibronectin expression. These findings establish ACLY as a promising target for genetic anti-fibrotic interventions (Kuwahara et al., 2025).

##### 5.3.1.4 Challenges and future directions

Although gene therapy brings hope for improving cardiac fibrosis, its clinical transformation faces a series of serious challenges. Gene therapies still lack an efficient and safe delivery system, and gene editing technology itself is accompanied by inherent risks. In terms of treatment strategy, whether it is over-expressing protective genes such as PGC1α and superoxide dismutase 2 or editing pathogenic targets such as proprotein convertase subtilisin/kexin type 9, we are faced with the problem of how to achieve accurate and controllable space-time expression to avoid interference with normal physiological functions. In addition, there are differences between animal models and human diseases. The same high AAV carrier dose as adult mice is used in newborn mice, but cardiac transduction is more effective in newborn mice. In clinical practice, we still need to further study the dose suitable for the human body (Doisne et al., 2021).

Therefore, the key to the future breakthrough is to develop new delivery tools with stronger heart specificity and higher security, and to create more accurate and controllable gene regulation technologies to give full play to the potential of gene therapy.

#### 5.3.2 Natural products and nutritional intervention

In addition to the synthetic drugs and gene therapies discussed above, several natural products have demonstrated anti-fibrotic effects through multi-target modulation of metabolic reprogramming. **Table 3** summarizes the mechanisms, experimental efficacy, and major limitations of representative natural products including resveratrol, curcumin, omega-3 fatty acids, vitamin D, and coenzyme Q10. As discussed in Section 4.3, the mechanisms by which these natural products modulate key signaling pathways (SIRT1/NF-κB, PI3K-AKT) are directly involved in the interactive activation of fibrotic signals (Walle, 2011; Liu et al., 2016; Liu et al., 2019; Roffe-Vazquez et al., 2019; Shabani et al., 2019; Xue et al., 2019; Zou et al., 2019; Hu et al., 2020; Saleh et al., 2020; Szeiffova Bacova et al., 2020; Thérien et al., 2021; Wen et al., 2023; Noonin and Thongboonkerd, 2024) (**Table 3**).

**Table 3:** Mechanisms, effects and limitations of natural product interventions in cardiac fibrosis.

Evidence level	Active substance	Mechanism	Effect	Limitations	References
High glucose-induced mouse model of renal fibrosis	curcumin	Inhibition of glycolysis, activation of FAO and restoration of OXPHOS to reverse metabolic reprogramming.	Inhibition of TGF-β secretion and collagen deposition to improve fibrosis.	Low bioavailability and inefficient intestinal absorption.	(Noonin and Thongboonkerd, 2024) (Liu et al., 2016)
Clinical studies in heart failure and animal models	vitamin D	Inhibition of hyperactivation of the renin-angiotensin-aldosterone system and the NF-κB pathway reduces transcription of inflammatory factors.	Reduces inflammation, regulates collagen expression and inhibits lipid accumulation processes.	High dose toxicity to the heart.	(Saleh et al., 2020) (Hu et al., 2020) (Roffe-Vazquez et al., 2019)
Animal models of fibrosis (TAC, ISO-induced)	resveratrol	Activation of SIRT1 and inhibition of the NF-κB pathway.	Reduces levels of myocardial inflammation, inhibits abnormal fibroblast activation, slows the progression of cardiac fibrosis, and improves cardiac diastolic function.	Low bioavailability.	(Zou et al., 2019) (Liu et al., 2019) (Walle, 2011)
Rat models of MI and spontaneous hypertension	omega-3 fatty acid	Replacement of omega-6 fatty acids to generate anti-inflammatory mediators.	Lower TG, reduce fibrosis.	Requires long-term supplementation.	(Shabani et al., 2019) (Szeiffova Bacova et al., 2020) (Thérien et al., 2021)
Animal models of pancreatic fibrosis and cellular experiments	coenzyme Q10	Inhibition of the PI3K-AKT pathway to reduce the production of inflammatory mediators and exert anti-lipid peroxidation effects.	Improves mitochondrial function and reduces inflammatory response.	Requires long-term supplementation.	(Wen et al., 2023) (Xue et al., 2019)

### 5.4 Emerging clinical translation directions

The preclinical findings described above highlight the therapeutic potential of targeting metabolic pathways to combat cardiac fibrosis. However, progress in translating these metabolic modulators into clinical applications remains limited, with most drug candidates still in the early stages of development. Nonetheless, anti−interleukin−11 (IL−11) antibodies have entered clinical evaluation for fibrotic diseases.

The role of IL-11 in fibrosis has been established in cardiac research. Animal studies have demonstrated that specific IL-11 transgenic expression or injection leads to cardiac fibrosis, while IL-11RA1 KO mice exhibit a lower incidence of cardiac fibrosis following AngII infusion compared to wild-type mice. Furthermore, IL-11 neutralizing antibodies can block fibroblast activation, all of which demonstrate that inhibiting IL-11 can reduce cardiac fibrosis (Schafer et al., 2017). The therapeutic effects of IL-11 have been extended to multiple organs: mouse model of pulmonary fibrosis and liver fibrosis (Ng et al., 2019) (Widjaja et al., 2019) (Dong et al., 2021). In humans, Boehringer Ingelheim is currently developing an anti-IL-11 antibody for the treatment of fibrosis. According to reports, Boehringer Ingelheim’s BI 765423 is a first-in-class anti-IL-11 antibody that has completed a Phase I evaluation in healthy volunteers (NCT05658107) and has entered Phase IIa clinical trials for the treatment of idiopathic pulmonary fibrosis.

Importantly, IL-11, as a key pro-fibrotic cytokine downstream of TGF-β, is increasingly recognized as a factor that directly drives the metabolic reprogramming of fibroblasts (Cook, 2023). IL−11 directly reprograms fibroblast metabolism via the ERK–HIF−1α axis, promoting glycolysis and inhibiting FAO, as shown in silicosis−induced pulmonary fibrosis and liver fibrosis (Dong et al., 2021) (Xie et al., 2024). Glutamine metabolism has also been identified as an upstream regulator of IL−11 expression (Chen et al., 2026b). Collectively, these findings indicate that metabolic reprogramming is not merely a consequence but a driver of fibrosis, and that IL−11 represents a novel metabolic−modulating anti−fibrotic target.

**Figure 3:**
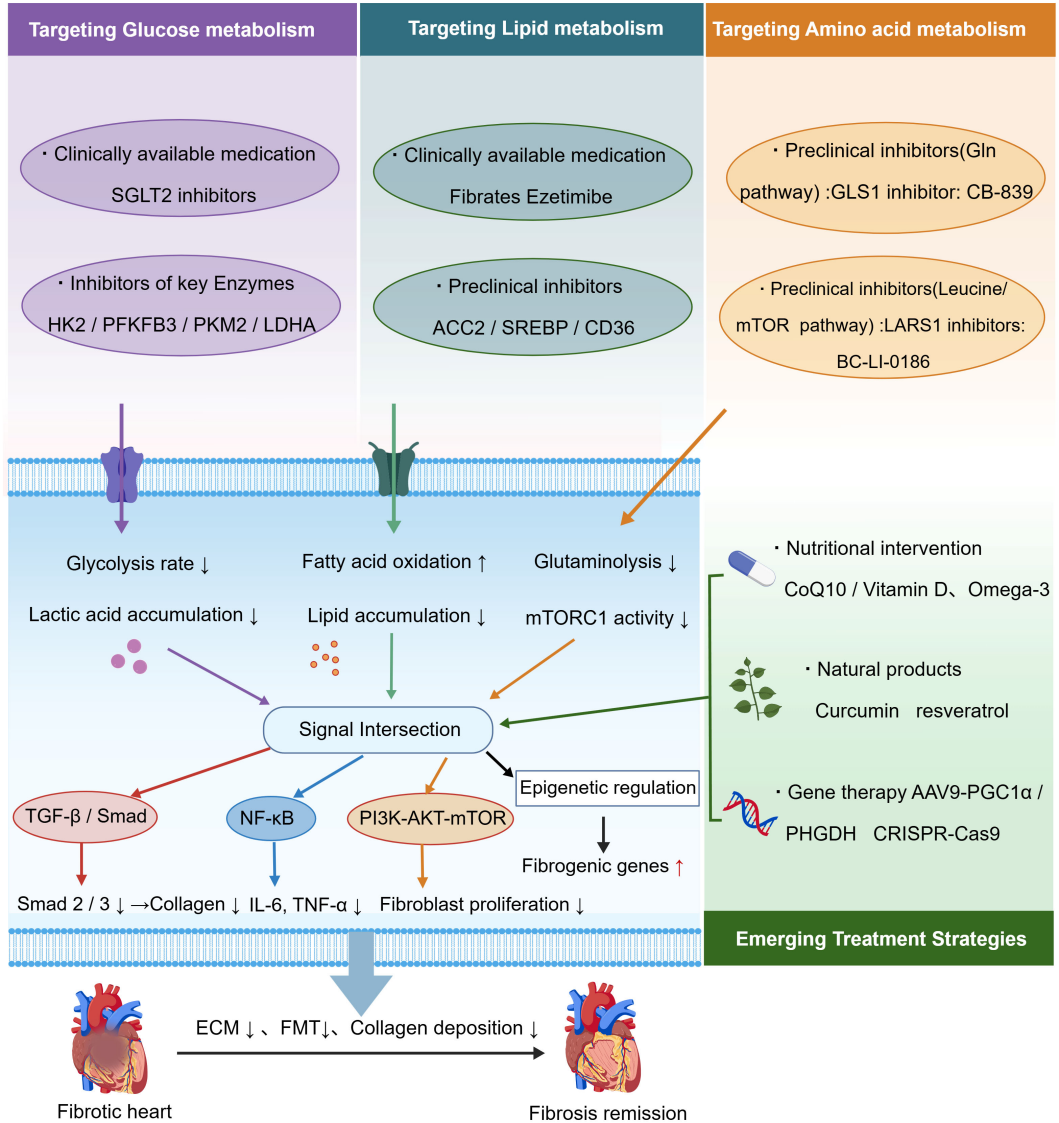
Therapeutic targeting of metabolic crosstalk in cardiac fibrosis. Anti-fibrotic interventions grouped by metabolic pathway. The signaling intersection indicates the anticipated therapeutic effects on key pathways, highlighting the crosstalk between metabolic pathways. Emerging strategies include gene therapy and natural products/nutritional interventions. ↑, increase/activation; ↓, decrease/inhibition. Created with BioGDP.com.

Therapeutic strategies targeting metabolic reprogramming in cardiac fibrosis are summarized in **Figure 3** (Jiang et al., 2025).

## 6 Conclusion and prospect

There is an inseparable relationship between the development of cardiac fibrosis and metabolic reprogramming. From the energy metabolic imbalance in the initial injury stage of myocardial cells to the metabolic characteristic changes of cell phenotyping in the fibrosis process, metabolic reprogramming has always run through the whole process of cardiac fibrosis. It not only drives the fibrosis process by regulating key links such as fibroblast activation, inflammatory response and ECM remodeling, but also forms a complex interaction network with mitochondrial dysfunction, epigenetic regulation and other mechanisms.

Although metabolic reprogramming shows the potential to regulate cardiac fibrosis, it still faces many challenges. Existing metabolic regulators generally have problems such as off-target effects caused by insufficient targeting specificity, extensive tissue distribution, and compensatory activation of metabolic pathways. The metabolic heterogeneity between different disease stages and cell subgroups has not been fully analyzed, resulting in a lack of accuracy in the intervention strategy. At the same time, the metabolic differences between preclinical research and the actual pathological environment, and the impact of individualized metabolic characteristics on the treatment response and other problems need to be solved urgently.

Future research should focus on building a multi-dimensional research system, such as the use of single-cell metabolomics combined with spatial transcriptome technology, the development of new targeted drugs, and the use of nano-delivery technology to improve treatment specificity. Through cross-scale and multidisciplinary collaborative innovation, the use of metabolic reprogramming research opens a new path for precise intervention for the prevention and treatment of cardiac fibrosis.

## Glossary

2-DG: 2-deoxy-D-glucose
3PO: 3-(3-pyridinyl)-1-(4-pyridinyl)-2-propen-1-one
3-PG: 3-phosphoglycerate
4-HNE: 4-hydroxynonenal
AAV: adeno-associated virus
ACC: acetyl coenzyme A carboxylase
ACLY: ATP-citrate lyase
AKT: protein kinase B
α-KG: α-ketoglutaric acid
ApoC-III: Apolipoprotein C-III
α-SMA: α-smooth muscle actin
ATGL: adipose tissue glycerol-3-phosphate lyase
ATP: adenosine triphosphate
BCAAs: branched-chain amino acids
β-OHB: β-hydroxybutyrate
CPT1: carnitine palmityl transferase 1
CRISPR: clustered regularly interspaced short palindromic repeats
COL1A1: Collagen type I alpha 1
COL1: Collagen type I
COLIII: Collagen type III
DAG: diacylglycerol
ECM: extracellular matrix
EndoMT: Endothelial-to-Mesenchymal Transition
ER: endoplasmic reticulum
ERK: extracellular signal-regulated kinase
ERS: endoplasmic reticulum stress
FAO: fatty acid oxidation
FASN: fatty acid synthase
FMT: fibroblast-to-myofibroblast transformation
Gln: glutamine
GLS: glutaminase
GPX4: glutathione peroxidase 4
GSH: glutathione
H3K27me3: trimethylation of lysine 27 in histone H3
HDL-C: high-density lipoprotein cholesterol
HIF-1α: hypoxia-inducible factor 1α
HK2: hexokinase 2
IL-11: interleukin-11
IL-6: interleukin-6
IRE1α: inositol-requiring enzyme 1α
KIC: 2-ketoisocaproate
KO: knockout
LARS1: leucyl-tRNA synthetase 1
LDL-C: low-density lipoprotein cholesterol
LDHA: lactate dehydrogenase A
MAPK: mitogen-activated protein kinase
MDA: malondialdehyde
MI: myocardial infarction
mTOR: mechanistic target of rapamycin
mTORC1: mechanistic target of rapamycin complex 1
NAD⁺: niacinamide adenine dinucleotide
NF-κB: nuclear factor kappa-B
OXPHOS: oxidative phosphorylation
PERK: PKR-like ER kinase
PFK1: phosphofructokinase 1
PFKFB3: phosphofructokinase-2/fructose-2,6-bisphosphatase 3
PGC1α: peroxisome proliferator-activated receptor gamma coactivator 1-alpha
PHGDH: phosphoglycerate dehydrogenase
PI3K: phosphatidylinositol 3-kinase
PKM2: pyruvate kinase isoform M2
PPARα: peroxisome proliferator-activated receptor alpha
PPARβ: peroxisome proliferator-activated receptor beta
ROS: reactive oxygen species
SCD1: sterol coenzyme A desaturase 1
SGLT2: sodium-glucose cotransporter 2
SIRT1: sirtuin 1
SIRT3: sirtuin 3
Smad2: SMAD family member 2
Smad3: SMAD family member 3
SREBP: sterol regulatory element binding protein
TAC: transverse aortic constriction
TCA: tricarboxylic acid
TG: Triglycerides
TGF-β: transforming growth factor-beta
SCOT: succinyl−CoA:3−ketoacid CoA transferase
